# Clarification of the Phylogenetic Framework of the Tribe Baorini (Lepidoptera: Hesperiidae: Hesperiinae) Inferred from Multiple Gene Sequences

**DOI:** 10.1371/journal.pone.0156861

**Published:** 2016-07-27

**Authors:** Xiaoling Fan, Hideyuki Chiba, Zhenfu Huang, Wen Fei, Min Wang, Szabolcs Sáfián

**Affiliations:** 1 Department of Entomology, College of Agriculture, South China Agricultural University, Guangzhou, China; 2 B. P. Bishop Museum, Honolulu, Hawaii, United States of America; 3 Institute of Silviculture and Forest Protection, University of West Hungary, Sopron, Hungary; Sichuan University, CHINA

## Abstract

Members of the skipper tribe Baorini generally resemble each other and are characterized by dark brown wings with hyaline white spots. These shared characteristics have caused difficulties with revealing the relationships among genera and species in the group, and some conflicting taxonomic views remain unresolved. The present study aims to infer a more comprehensive phylogeny of the tribe using molecular data, to test the monophyly of the tribe as well as the genera it includes in order to clarify their taxonomic status, and finally to revise the current classification of the group. In order to reconstruct a phylogenetic tree, the mitochondrial *COI-COII* and *16S* genes as well as the nuclear *EF-1α* and *28S* genes were analyzed using parsimony, maximum likelihood, and Bayesian inference. The analysis included 67 specimens of 41 species, and we confirmed the monophyly of Baorini, and revealed that 14 genera are well supported. The genus *Borbo* is separated into three clades: *Borbo*, *Pseudoborbo*, and *Larsenia* gen. nov. We confirmed that *Polytremis* is polyphyletic and separated into three genera: *Polytremis*, *Zinaida*, and *Zenonoida* **gen. nov**., and also confirmed that the genus *Prusiana* is a member of the tribe. Relationships among some genera were strongly supported. For example, *Zenonia* and *Zenonoida* were found to be sister taxa, closely related to *Zinaida* and *Iton*, while *Pelopidas* and *Baoris* were also found to cluster together.

## Introduction

The family Hesperiidae, commonly known as skippers or skipper butterflies, comprises approximately 4000 species belonging to 540 genera worldwide [1] and is defined by the following unique morphological character states: an “eye ring”, a wide head, an area of small and specialized scales on the upper side of the hindwing base, and a large thorax, resulting in the mesoscutellum overhanging the metanotum [2]. These unique character states support monophyly of the family [2–4], which has also been supported by molecular data [5]. The higher classification of Hesperiidae had long remained unchanged until Warren *et al*. challenged it with phylogenetic scheme [2, 5]. The traditional framework of six subfamilies was rearranged into seven: Coeliadinae, Euschemoninae, Eudaminae, Pyrginae, Heteropterinae, Trapezitinae, and Hesperiinae. Moreover, they reinterpreted Evans’ [6] genus groups as tribes, and furthermore the subfamily Hesperiinae was subdivided into six tribes, and the tribe Baorini was proposed [5]. Monophyly of the tribe was strongly supported by both a study using only molecular data [5] as well as another that combined molecular and morphological data [2].

The tribe Baorini was originally introduced as Baorina, one of the subfamilies of Hesperiadae [sic] [7]. The subfamily level designation was also used by Bell [8] in which *Baoris* Moore, 1881, *Caltoris* Swinhoe, 1893, *Chapra* Moore, 1881 (a junior subjective synonym of *Pelopidas* Walker, 1870), *Parnara* Moore, 1881, *Gegenes* Hübner, 1819, and *Iton* de Nicéville, 1895 were included. Evans [6] placed these genera in his Gegenes group. Subsequently, Eloit named it the Pelopidas group [9, 10], and Chou employed the tribe name Gegenini [11, 12]. According to Code article 23.3.1 and 34.1 of International Commission on Zoological Nomenclature [13], the designation Baorini is more appropriate than Gegenini.

Most members of the group resemble each other and have dark brown wings and hyaline white spots. Mainly due to this simple wing pattern, researchers have struggled to determine which species were related and should be assigned to the same genus. In the renowned *Die Gross Schmetterlinge der Erde*, all members, except for *Gegenes*, were placed in *Parnara* [14–16]. Simultaneously, *Zenonia zeno* was considered a species of *Padraona*, whose markings are orange and yellow. It is worth noting that Mabille described the genus *Polytremis* in 1904 [17], but this description was not reflected in a later publication in 1909 [14]. Evans [18, 19] also initially published contradictory definitions of the taxonomy within the group. Initially, he assigned almost all species to *Baoris*, except for some that were placed in the genera *Iton* and *Gegenes*, even though all of the major genera had been previously described. Evans worked extensively on the group until he finally settled on eight genera [6].

In most current taxonomic studies, the six genera mentioned above as well as two African allies, *Zenonia* and *Brusa*, are treated as members of the same group, regardless of the name used. However, *Prusiana*, *Pseudoborbo*, and *Zinaida* are exceptions and further explanation clarifying why they are distinct is necessary.

Although Evans recognized that the genitalia of the genus *Prusiana* were the same as the Gegenes group, he still treated the genus as a member of his Taractrocera group and placed it after the genus *Cephrenes* [6]. De Jong considered *Prusiana* to be a rather enigmatic group due to its unclear relationship to other genera [20]. Maruyama, regarded the difference in genitalia morphology to be an important taxonomic character, and moved the genus into the Pelopidas group [21], which is currently generally followed in classification schemes [2, 22].

*Hesperia bevani* Moore, 1878 was assigned previously to various genera, such as *Baoris* [23–26], *Parnara* [14, 16, 27–38], *Caltoris* [39, 40], or *Pelopidas* [19]. Since Evans described the genus *Borbo* [6], this species is usually placed in this genus [10, 22, 41–44]. Subsequently, Lee described the genus *Pseudoborbo* based on the adult and immature morphological characters of *Borbo bevani* and then reclassified this species as his monotypic genus [45]. Some subsequent authors, however, did not support Lee’s arrangement and considered the genus *Pseudoborbo* to be a synonym of *Borbo* [2, 5, 46, 47], while others followed Lee’s classification [1, 11, 12, 48–52].

The genus *Zinaida* was described by Evans with *Parnara nascens* Leech, 1893 as its type species. In addition to the type species, *Z*. *theca* Evans, 1937 was described and *Pamphila caerulescens* Mabille, 1876 and *Pamphila mencia* Moore, 1877 were also included in the genus [19]. Without any explanation, however Evans treated *Zinaida* as a synonym of the genus *Polytremis* Mabille, 1904 [6]. Subsequent authors also followed this classification scheme [1, 10–12, 41, 42, 52].

Few phylogenetic analyses involving the tribe Baorini have been published. Dodo *et al*. analyzed mitochondrial *ND5* and *COI* of Japanese skippers, and concluded that the genera *Pelopidas* and *Parnara* were monophyletic groups [53], which we have confirmed in this study. Warren *et al*. investigated the phylogenetic relationships of subfamilies and the circumscription of tribes of the family Hesperiidae based on molecular data [5]. Baorini included only four species belonging to three genera—*Pelopidas*, *Iton*, and *Polytremis*—and it was concluded that the monophyly of the Baorine clade was strongly supported. Warren *et al*. used 49 morphological characters and molecular data to revise the classification of the family Hesperiidae and confirmed the robust monophyly of the tribe Baorini [2], although, only the above three genera were included. A molecular phylogenetic study of Chinese skippers, which sampled only six species across three genera (*Parnara*, *Pelopidas*, and *Polytremis*), provided evidence that the tribe is monophyletic [54].

Jiang *et al*. constructed a phylogeny of the genus *Polytremis* from China using one mitochondrial and two nuclear derived genes and claimed that the monophyly of the genus was supported [55]. Yuan *et al*. analyzed three mitochondrial genes of three species from China, but could not confirm these findings [54]. Our results also contradict the conclusions made by Jiang *et al*. [55].

The objectives of the present study were to infer a more comprehensive phylogeny of the tribe Baorini using molecular data, to test the monophyly of the tribe Baorini, to clarify the taxonomic status of multiple genera, and to revise the current classification within this tribe if necessary. A well-resolved phylogeny of the tribe Baorini will enhance the understanding of the evolution and biology among species within this group.

## Materials and Methods

### Taxon sampling

Samples were obtained from all major genera in the tribe Baorini except for *Brusa*. When possible, the type species was included and multiple species were chosen in controversial genera to correctly clarify taxonomic status. In total, 67 specimens representing 41 species across 11 genera of the tribe Baorini were selected as ingroup taxa. Specifically, we included the genus *Pseudoborbo*, which has been considered a synonym of *Borbo* by some authors; *Prusiana*, which was considered a member of Taractrocera group [6]; and *Polytremis nascens*, the type species of the genus *Zinaida*, believed to be a synonym of *Polytremis*. An additional six species, including single representatives from two genera of the Taractrocerini tribe, *Taractrocera* and *Telicota*, as well as the genera *Aeromachus*, *Ampittia*, *Daimio*, and *Tagiades* were used as outgroups to assess the status of the genus *Prusiana* and the stability of basal relationships among ingroup lineages. Voucher specimens representing all sampled species were deposited in the Insect Collection of the South China Agricultural University (SCAU). Specimen information and location data are presented in Table 1.

**Table 1:** Species information and GenBank accession numbers.

Taxon	Locality	Voucher	The type species	GenBank Accession Nos.			
				16S	COI-COII	28S	EF-1a
*Parnara guttata* (Bremer & Grey, 1853) 1	China: Guangdong, Yingde	He001	●	JX971164	JX989082	JX989114	KX151612
*Parnara guttata* (Bremer & Grey, 1853) 2	China: Guangdong, Yingde	He003	●	JX971165	JX989083	JX989115	KX151613
*Parnara ganga* Evans, 1937 1	China: Hainan, Jianfengling	He028		JX971166	JX989084	JX989116	KX151609
*Parnara ganga* Evans, 1937 2	China: Hainan, Jianfengling	He029		JX971167	JX989085	JX989117	KX151610
*Parnara ganga* Evans, 1937 3	China: Hainan, Jianfengling	He030		JX971168	JX989086	JX989118	KX151611
*Parnara bada* (Moore, 1878)	China: Guangxi, Maoershan	He012		JX971169	JX989087	JX989119	KX151608
*Polytremis lubricans* (Herrich-Schäffer, 1869) 1	China: Guangdong, Nanling	He095	●	JX971170	JX989088	JX989120	KX151619
*Polytremis lubricans* (Herrich-Schäffer, 1869) 2	China: Hainan, Jianfengling	He160	●	JX971171	JX989089	JX989121	KX151620
*Polytremis lubricans* (Herrich-Schäffer, 1869) 3	Malaysia: Perak,Kinta Highland	He549	●	KX151512	KX151572	-	KX151621
*Polytremis lubricans* (Herrich-Schäffer, 1869) 4	Malaysia, Perak,Kinta Highland	He550	●	KX151513	-	KX151545	KX151622
*Polytremis lubricans* (Herrich-Schäffer, 1869) 5	Malaysia, Perak,Kinta Highland	He551	●	KX151514	KX151573	KX151546	KX151623
*Polytremis caerulescens* (Mabille, 1876)	China: Sichuan, Luding, Moxi	He087		JX971172	JX989090	JX989122	KX151616
*Polytremis zina zina* (Evans, 1932)	China: Guangdong, Nanling	He037		JX971173	JX989091	JX989123	KX151631
*Polytremis zina taiwana* Murayama, 1981	Taiwan	He545		KX151519	KX151578	KX151551	KX151632
*Polytremis theca theca* (Evans, 1937)	China: Shaanxi, Qinling	He503		KX151518	KX151577	KX151550	KX151630
*Polytremis theca fukia* Evans, 1940	China: Guangdong, Nanling	He009		JX971174	JX989092	JX989124	KX151629
*Polytremis suprema* Sugiyama, 1999	China:Guangdong, Nanling	He070		JX971175	JX989093	JX989125	KX151628
*Polytremis nascens* (Leech, 1893)	China: Sichuan, Baoxing	He100	●	JX971176	JX989094	JX989126	KX151626
*Polytremis gotama* Sugiyama, 1999	China: Yunnan, luguhu	He010		JX971177	-	JX989127	-
*Polytremis mencia* (Moore, 1878)	China: Jiangxi, Lushan	He502		KX151516	KX151575	KX151548	KX151625
*Polytremis matsuii* Sugiyama, 1999	China: Sichuan, Hailuogou	He484		KX151515	KX151574	KX151547	KX151624
*Polytremis pellucida* (Murray, 1874)	Janpan: Kumamoto	He392		KX151517	KX151576	KX151549	KX151627
*Polytremis discreta* (Elwes & Edwards, 1897) 1	Vietnam: Dac Lae, Chu Yang Sin	He447		KX151506	-	KX151539	-
*Polytremis discreta* (Elwes & Edwards, 1897) 2	China: Sichan, Hanyuan	He448		KX151507	-	KX151540	-
*Polytremis discreta* (Elwes & Edwards, 1897) 3	China: Sichuan, Yaan	He481		KX151508	KX151570	KX151541	KX151617
*Polytremis eltola* (Hewitson, 1869) 1	China: Hunan, Mangshan	He104		KX151509	-	KX151542	-
*Polytremis eltola* (Hewitson, 1869) 2	Vietnam:Dac Lae, Chu Yang Sin	He446		KX151510	-	KX151543	-
*Polytremis eltola* (Hewitson, 1869) 3	China: Hunan, Mangshan	He509		KX151511	KX151571	KX151544	KX151618
*Borbo borbonica* (Boisduval, 1833)	Kenya: Embu	JS064	●	KX151490	KX151557	KX151525	-
*Borbo cinnara* (Wallace, 1866) 1	China: Guangdong, Nanling	He017		JX971178	JX989095	JX989128	KX151587
*Borbo cinnara* (Wallace, 1866) 2	China: Hainan, Jianfengling	He017’		JX971179	JX989096	JX989129	KX151588
*Borbo fatuellus* (Hopffer, 1855) 1	Liberia: Nimba mountains	Tok17		KX151492	KX151559	KX151527	-
*Borbo fatuellus* (Hopffer, 1855) 2	Liberia: Nimba mountains	VA35		KX151491	KX151558	KX151526	KX151589
*Borbo gemella* (Mabille, 1884)	Liberia: Nimba mountains	GA13		KX151493	KX151560	KX151528	KX151590
*Borbo holtzi* (Plötz,1883) 1	Liberia: Nimba mountains	VA24		KX151494	KX151561	-	KX151591
*Borbo holtzi* (Plötz,1883) 2	Liberia: Nimba mountains	VA40		KX151495	KX151562	-	-
*Borbo perobscura* (Druce, 1912)	Liberia: Nimba mountains	GA8		KX151496	KX151563	KX151529	-
*Borbo ratek* (Boisduval, 1833) 1	Madagascar	JS071		KX151497	KX151564	KX151530	KX151592
*Borbo ratek* (Boisduval, 1833) 2	Madagascar	SZS-BOR-004		KX151498	KX151565	KX151531	KX151593
*Borbo ratek* (Boisduval, 1833) 3	Madagascar	SZS-BOR-006		KX151499	-	KX151532	KX151594
*Borbo* sp.	Liberia: Nimba mountains	GA19		KX151500	KX151566	KX151533	KX151595
*Pseudoborbo bevani* (Moore, 1878)	China: Guangdong, Yingde	He018	●	JX971180	JX989097	JX989130	KX151633
*Pelopidas mathias* (Fabricius, 1798)	China: Fujian	He194		JX971181	JX989098	JX989131	KX151615
*Pelopidas agna* (Moore, 1866)	China: Hainan, Jianfengling	He013		JX971182	JX989099	JX989132	KX151614
*Pelopidas thrax* Hübner,1821*	Ghana: Ashanti Region		●	-	EU364491*	-	EU364286*
*Caltoris bromus* (Leech, 1893) 1	China: Guangdong, Yingde	He002		JX971183	JX989100	JX989133	KX151596
*Caltoris bromus* (Leech, 1893) 2	China: Guangxi, Maoershan	He024		JX971184	JX989101	JX989134	KX151597
*Caltoris bromus* (Leech, 1893) 3	China: Guangxi, Maoershan	He025		JX971185	JX989102	JX989135	KX151598
*Caltoris bromus* (Leech, 1893) 4	China: Hainan, Jianfengling	He032		JX971186	JX989103	JX989136	KX151599
*Caltoris cahira* (Moore, 1878) 1	China: Guangxi, Maoershan	He022		JX971187	JX989104	JX989137	KX151601
*Caltoris cahira* (Moore, 1878) 2	China: Guangxi, Maoershan	He023		JX971188	JX989105	JX989138	KX151602
*Caltoris kumara* (Moore, 1878)	Java: Mt. Pagoberan	He540	●	KX151504	KX151569	KX151537	KX151605
*Caltoris malaya* (Evans, 1926)	Malaysia: Perak	He541		KX151505	-	KX151538	KX151606
*Caltoris brunnea* (Snellen, 1876)	Java: Mt. Pagoberan	He542		KX151501	KX151567	KX151534	KX151600
*Caltoris cormasa* (Hewitson, 1876) 1	Java: Mt. Pagoberan	He543		KX151502	-	KX151535	KX151603
*Caltoris cormasa* (Hewitson, 1876) 2	Malaysia: Perak	He544		KX151503	KX151568	KX151536	KX151604
*Baoris farri* (Moore, 1878) 1	China: Hainan, Jianfengling	He091		JX971189	JX989106	JX989139	KX151584
*Baoris farri* (Moore, 1878) 2	China: Guangdong, Guangzhou	He049		JX971190	JX989107	JX989140	-
*Baoris penicillata* (Moore, 1881)	China: Hainan, Yinggeling	He112		JX971191	JX989108	JX989141	KX151586
*Baoris leechii* (Elwes & Edwards, 1897)	China: Guangdong, Nanling	He093		KX151489	-	KX151524	KX151585
*Iton semamora* (Moore, 1866)	Indonesia: Sumatra	He239	●	JX971192	JX989109	JX989142	KX151607
*Iton watsonii* (de Nicéville, 1890)	Thailand: Chiang Mai			-	EU364490*	-	EU364285*
*Gegenes nostrodamus* (Fabricius, 1793)	Morocco: Marrakech	He240		JX971193	-	JX989143	-
*Prusiana prusias* matinus (Fruhstorfer, 1911) 1	Philippines: Leyte	He241	●	JX971194	-	JX989144	-
*Prusiana prusias* matinus (Fruhstorfer, 1911) 2	Philippines: C. Palawan	He393	●	KX151520	KX151579	KX151552	KX151634
*Zenonia zeno* (Trimen, 1864) 1	Cameroon: N. Cameroon	SZS-ZEN-001	●	KX151521	KX151580	KX151553	KX151635
*Zenonia zeno* (Trimen, 1864) 2	Kenya: Nairobi	SZS-ZEN-002	●	-	KX151581	KX151554	-
*Aeromachus stigmatus* (Moore, 1878)	China: Yunnan, Hutiaoxia	He434	●	KX151522	KX151582	KX151555	KX151636
*Ampittia virgata* Leech, 1890	China: Guangdong, Nanling	He008		KX151523	KX151583	KX151556	KX151637
*Telicota augias*	China: Guangdong, Yingde	He082		JX971195	JX989110	JX989145	KX151638
*Potanthus trachala*	China: Guangxi, Guiling	He346		JX971196	JX989111	JX989146	KX151639
*Tagiades menaka*	China: Guangdong, Yingde	He004		JX971197	JX989112	JX989147	KX151640
*Daimio tethys*	China: Guangxi, Maoershan	He384		JX971198	JX989113	JX989148	KX151641

* GenBank accession numbers correspond to specimens in Warren *et al*. (2008).

### DNA extraction, PCR amplification, and sequencing

Total genomic DNA was extracted from the thorax of specimens preserved in ethanol, or from one to three legs of dried specimens. The tissues were macerated in 500 μL Proteinase K solution (10 mM Tris HCl, 10 mM EDTA, 150 mM NaCl, and 0.5 mg/mL proteinase K), and incubated at 55°C for 2–3 h. The resulting solution was extracted once with phenol saturated with TE buffer (10 mM Tri-HCl [pH 8.0] and 1 mM EDTA), once with phenol/chloroform (1:1), and once with chloroform/isoamyl alcohol (24:1). The total DNA was precipitated by adding twice the volume of 100% ethanol and one-tenth the volume of 3 M sodium acetate to the supernatant, washed with 70% ethanol, dried, and then dissolved in 80–100 μL TE buffer. DNA from *Pseudoborbo bevani*, *Iton semamora*, *Prusiana kuehni*, and *Zenonia zeno* specimens was extracted from legs using the Qiagen DNeasy Blood and Tissue Kit (Qiagen, Hilden, Germany) following the manufacturer’s protocol for animal tissue.

Four target regions were amplified by PCR using the primers listed in Table 2. PCR reactions were performed in 25 μL volumes containing 2.5 μL 10×PCR buffer (2.0 mM MgCl2), 2 μL dNTPs (containing 2.5 mM of each dNTP), 1 μL of each primer (10 pmol/μL), 1 μL of template DNA, and 1.25 units Taq DNA polymerase (Takara Inc, Shiga, Japan). The amplification cycle was 95°C for 5 min, and for the *16S* and *28S* genes was followed by 35 cycles of 94°C for 30 sec, then 47°C (16S) or 50°C (28S) for 30 sec and 72°C for 1.5 min. For the *COI-COII* and *EF-1α*, the initial 95°C at 5 min was followed by 35 cycles of 94°C for 1 min, 46°C (*COI*-*COII*) or 55°C (*EF-1α*) for 1 min and 72°C for 2 min. All amplification cycles included a final extension period of 72°C for 7 min. Successful amplification was verified using agarose gel electrophoresis.

**Table 2:** Primers used for amplification and sequencing in this study.

Gene	Primer	Sequence (5′–3′)	Reference
COI-COII	Gary	TAGGAATAATTTATGCMATAATAGC	Warren *et al*.[5]
	Susan	TTGTTGTTCTAATARAAATCG	Warren *et al*.[5]
16S rRNA	LR-J-12887	CCGGTTTGAGCTCAGATCA	Simon *et al*.[56]
	LR-N-13398	CGCCTGTTTATCAAAAACAT	Simon *et al*.[56]
EF-1α	ef44	GCYGARCGYGARCGTGGTATYAC	Monteiro and Pierce [57]
	efrcM4	ACAGCVACKGTYTGYCTCATRTC	Monteiro and Pierce[57]
28S rRNA	28S-01	GACTACCCCCTGAATTTAAGCAT	Kim *et al*.[58]
	28SR-01	GACTCCTTGGTCCGTGTTTCAAG	Kim *et al*.[58]

PCR products were purified with a Gel DNA purification kit (Takara Inc), and were directly sequenced with the same primers listed in Table 2, or cloned and then sequenced. For cloning, the purified PCR products were cloned into the pMD18-T vector (Takara Inc) using *Escherichia coli* TG-1 as the host. At least three positive clones were selected for sequencing to correct for PCR errors. Sequencing was performed using the ABI 3730 automated sequencer. DNA sequences were assembled and edited with SeqManII in the DNASTAR package (DNASTAR Inc, Wisconsin, USA) and checked manually. All sequences were deposited in GenBank, and the accession numbers for each sequence are listed in Table 1.

### Data analyses

Alignments of the rRNA gene sequences were conducted with MAFFT (version 7) using separate gene partitions (*16S* and *28S*) via the online sever (http://mafft.cbrc.jp/alignment/server/). We used the Q-INS-I strategy, which accounts for the secondary structure of the RNA and small data sets (with less than 200 sequences), and ‘1PAM/κ = 2’, which is recommended for aligning closely related DNA sequences and the offset was set at 0.1 when large gaps were not expected based on preliminary analyses [59–61]. Both the *COI-COII* (only one 3-bp gap) and *EF-1α* sequences were aligned using the Clustal X [62] with the default settings. All base frequencies and molecular character statistics were calculated using MEGA 6.0 [63]. Homogeneity of the base frequencies across taxa was tested using the Chi-square test implemented in PAUP* 4.0b10 [64]. The incongruence length difference (ILD) test [65] in PAUP* was conducted to evaluate the congruence of mitochondrial (*COI-II* and *16S*) and nuclear (*EF-1α* and *28S*) markers and determine if they could be analyzed together. Only taxa with sequence information for all four target regions were included in this analysis. Saturation for each gene and for the codon positions of *COI*, *COII*, and *EF-1α* were assessed using the substitution saturation test [66, 67] in the program DAMBE [68].

Phylogenetic trees were constructed using the maximum parsimony (MP), maximum Likelihood (ML), and Bayesian inference (BI) methods. MP analyses were conducted using TNT version 1.1 [69] with the following options: parsimony-informative characters were unordered and equally weighted, gaps were treated as missing data, searches heuristic using a “driven search” until the minimum length was hit ten times by means of a combination of TreeFusion, Ratchet, Tree Drifting, and Sectorial searches under default parameters [70]. Branch support was assessed using the bootstrap test [71] with 1000 replicates.

Prior to ML and BI analyses, the best-fit model of nucleotide substitution was selected using jModeltest 2.1.7 [72] for each gene region (*COI* (GTR+I+G), *tRNAleu* (HKY+I), *COII* (GTR+I+G), *16S* (GTR+I+G), *EF-1α* (SYM+I+G), and *28S* (GTR+I+G)), and by codon position for *COI*, *COII* and *EF-1α* (seven partitions: 1st+2nd (GTR+I+G) and 3rd codon positions (GTR+I+G) of the mitochondrial protein coding genes *COI* and *COII* together, same for the nuclear gene *EF-1α* (positions (1+2): SYM+I+G, position 3: GTR+I+G), the mitochondrial RNA genes *tRNAleu* and *16S*, and also the nuclear *28S* gene) under the Akaike Information Criterion [73].

ML analyses were carried out using RAxML version 8 [74] on a concatenated data set of all genes, with 1000 rapid bootstraps using both GTR+G and GTR+I+G. The topologies of the trees were consistent, and support values for the clades only differed slightly. Here, we have only presented the results from the analysis using the GTR+G model. Bayesian analyses were conducted using MrBayes 3.2.2 [75] using the best-fit model determined using the two above-mentioned schemes. Four simultaneous chains were run for 5×10^6^ generations, and trees were sampled every 100 generations with the first 25% of sampled trees discarded as burn-in. The convergence of the analyses was determined with the program Tracer v1.6 [76] and Bayesian posterior probabilities were used to evaluate branch support. Both MrBayes and RAXML runs were carried out on the online CIPRES Science Gateway resource [77].

Bootstrap support values (BP, for MP; BS, for ML) and posterior probabilities (PP for BI) were used to assess the robustness of the results. In order to discuss the results, we have delimited the support values as strongly, moderately, and weakly supported. In the MP and ML analyses, we regard clades with bootstrap values of 69 and below to be weakly supported, 70–89 to be moderately supported, and 90 and above to be strongly supported. In the BI analyses, we considered clades with posterior probabilities of 0.79 and below to be weakly supported, those with probabilities of 0.80–0.94 to be moderately supported, and those with probabilities of 0.95 and above to be strongly supported.

### Nomenclature Acts

The electronic edition of this article conforms to the requirements of the amended International Code of Zoological Nomenclature, and hence the new names contained herein are available under that Code from the electronic edition of this article. This published work and the nomenclatural acts it contains have been registered in ZooBank, the online registration system for the ICZN. The ZooBank LSIDs (Life Science Identifiers) can be resolved and the associated information viewed through any standard web browser by appending the LSID to the prefix“http://zoobank.org/”. The LSID for this publication is: urn:lsid:zoobank.org:pub: 89BFF498-46F3-4007-87A9-F826290724C7. The electronic edition of this work was published in a journal with an ISSN, and has been archived and is available from the following digital repositories: PubMed Central, LOCKSS.

## Results

### Sequence Characteristics

From a total of 71 samples, we obtained 60, 70, 58, and 69 sequences for *COI-COII*, *16S*, *EF-1α* and *28S* sequences, respectively. In addition, we included an additional four sequences from two species from GenBank (Table 1).

The *COI-COII* (929 bp) region was composed of 703 bp of the *COI* gene, the entire 70 bp of the intervening *tRNAleu* (including one 3-bp gap since *Pelopidas agna* has a three-base-pair insertion), and 156 bp of the *COII* gene. Due to several small indels in some species, the *16S* and *28S* sequence lengths varied between 512–520 bp and 825–840 bp, respectively. In total, the alignment of the four regions consisted of a total of 3380 bp (929, 531, 1066, and 854 bp of the *COI-COII*, *16S*, *EF-1α* and *28S* genes, respectively), of which 975 positions were variable, and 747 were parsimony-informative. We failed to obtain sequences for some specimens, and the missing data were designated as a ‘?’ in the alignment. Within the ingroup, average base composition was T = 30.4%, C = 21.1%, A = 28.8%, and G = 19.7%. The Chi-square test revealed no significant base composition heterogeneity across samples employed (df = 150, P = 1.00). For all three codon positions of *COII* and *EF-1α* as well as for the three regions *tRNAleu*, *16S*, and *28S*, the value of the substitution saturation index (*I*_*ss*_) was much smaller than the critical value (*I*_*ss*. *c*_), assuming either a symmetrical topology or an asymmetrical topology. These results show that these data subsets are unlikely to have reached saturation. For *COI*, only the third codon position reveals that *I*_*ss*_ is larger than *I*_*ss*.*c*_, assuming an asymmetrical topology. Therefore, there is little substitution saturation in our sequence data.

The ILD test revealed no significant incongruence between the two data sets (mtDNA *COI-II* and *16S* vs. rDNA *EF-1α* and *28S*, P = 0.19), indicating that the sequences could be combined in the phylogenetic reconstruction.

### Phylogenetic analyses

**Fig 1:**
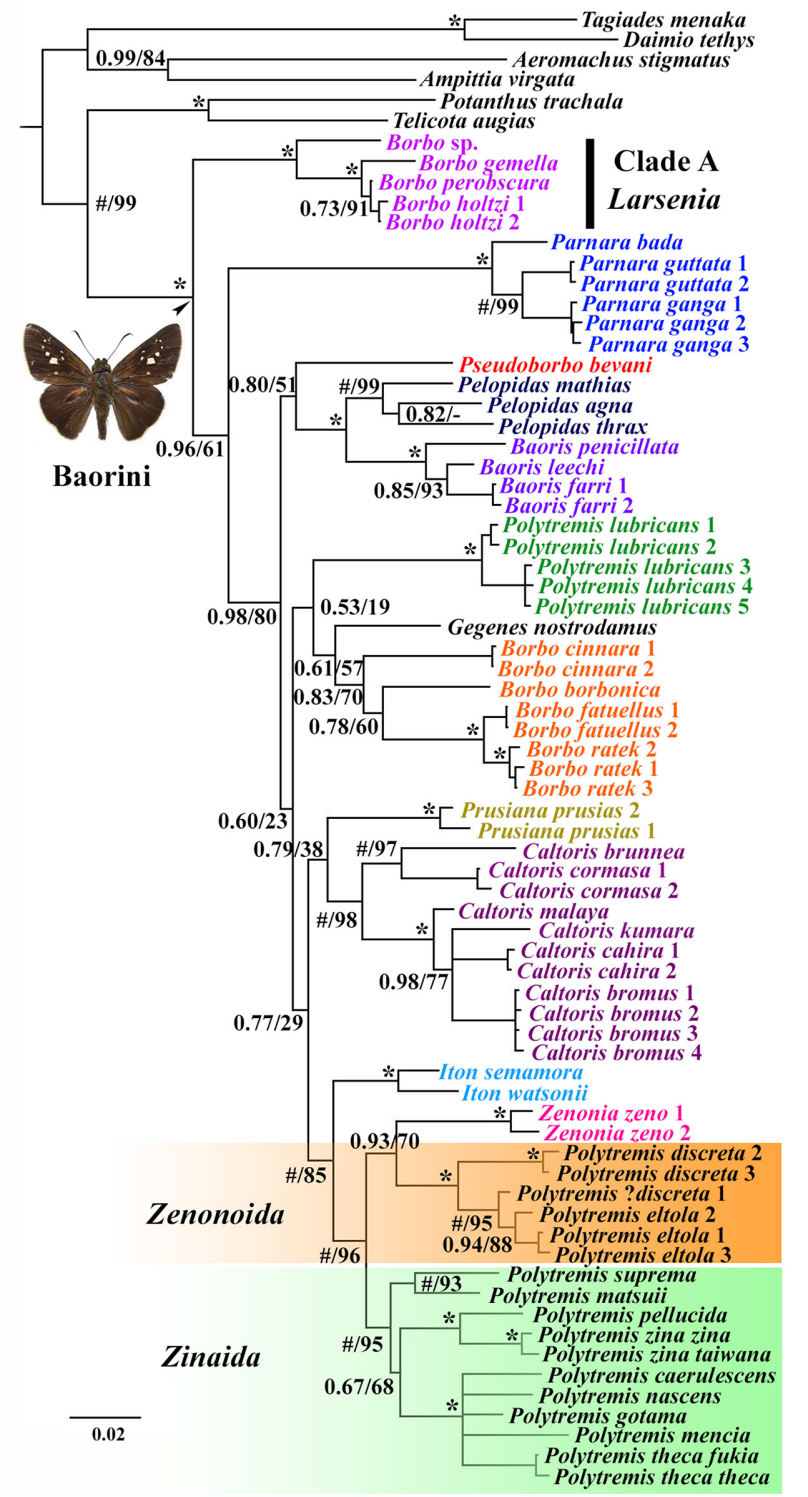
Majority-rule consensus tree from the Bayesian analysis (BI) of the concatenated *COI-COII*, *16S*, *EF-1α*, and *28S* sequences. Values at nodes represent the posterior probabilities (PP) of BI and the bootstrap support (BS) values of the maximum likelihood (ML) analysis, respectively. Asterisks indicate branches supported 100% by both PP and BS. “#” indicates that PP = 100. Colors highlight recognized genera.

The three concatenated analyses (BI, ML, and MP) revealed similar topologies, differing mainly in branch support (Fig 1, S1 Fig); however, the monophyly of the tribe Baorini is strongly supported in all methods (PP = 1.00, BS = 100, BP = 100). Within the tribe, although support for some basal clades is low, the monophyly of the seven traditionally established genera (*Parnara*, *Pelopidas*, *Baoris*, *Caltoris*, *Prusiana*, *Iton*, and *Zenonia*) is strongly supported in all phylogenetic analyses. On the other hand, contrary to conventional taxonomy, the genera *Borbo* and *Polytremis* are not monophyletic. Members of *Borbo* did not form a cluster, but instead formed three clades—Clade A, the *Borbo* clade, and the *Pseudoborbo* clade (which only included the species *P*. *bevani*, which was previously placed within *Borbo*). Clade A is a strongly supported monophyletic group (PP = 1.00, BS = 100, BP = 100) that consists of the following species: *B*. sp., *B*. *gemella*, *B*. *holtzi*, and *B*. *perobscura* and is, by this analysis, sister to the other remaining Baorini. We designated the clade to have a new genus status, *Larsenia* gen. nov. The genus *Pseudoborbo* has a controversial taxonomic status and according to all of the methods is sister to *Pelopidas* and *Baoris*, which is moderately supported in BI analysis (PP = 0.80). We determined that *Pelopidas* is sister to *Baoris* (PP = 1.00, BS = 100, BP = 97). The genus *Borbo*, excluding *Larsenia* and *Pseudoborbo*, was moderately supported in the BI and ML analyses (PP = 0.83, BS = 70). For the genus *Polytremis*, all members analyzed here except for *P*. *lubricans*, together with the genus *Zenonia*, formed a strongly supported monophyletic clade (PP = 1.00, BS = 96, BP = 91), which is sister to the genus *Iton* (PP = 1.00, BS = 85, BP = 65). Within the clade, *P*. *eltola* and *P*. *discreta* formed a strongly supported monophyletic group (PP = 1.00, BS = 100, BP = 100), which is sister to the genus *Zenonia*, with moderate support (PP = 0.93, BS = 70, BP = 66). We recognized the *P*. *eltola* and *P*. *discrete* clade to have a new genus status. Other species of *Polytremis* sensu Evans [6] including *P*. *nascens* (the type species of *Zinaida*) appeared to form a monophyletic group with strong support (PP = 1.00, BS = 95, BP = 81). *P*. *lubricans*, the type species of *Polytremis*, formed a separate clade from other *Polytremis* sensu Evans [6] species. Consequently, we propose that the genus *Zinaida* Evans, 1937 be reinstated. Based on highly supported monophyly of these genera, together with morphological characters, herein we have designated the following fourteen clades as genera: *Larsenia* **gen. nov**., *Parnara*, *Gegenes*, *Borbo*, *Pelopidas*, *Baoris*, *Caltoris*, *Pseudoborbo*, *Polytremis*, *Prusiana*, *Iton*, *Zenonia*, *Zenonoida* **gen. nov**., and *Zinaida*.

## Discussion

Although the basal relationships within Baorini were poorly resolved, proximal clades were strongly supported across all analyses. Of the 14 major lineages we defined here as genera, eight (*Parnara*, *Gegenes*, *Pelopidas*, *Baoris*, *Caltoris*, *Prusiana*, *Iton*, and *Zenonia*) are concordant with traditionally established genera, while the others are inconsistent with the previously described genera.

### *Larsenia* Chiba, Fan & Sáfián gen. nov.

urn:lsid:zoobank.org:act:E3CA9226-4199-48BC-9D92-7A1A3F293E49

#### Type species. *Hesperia holtzi* Plötz, 1883 Male [78]

Diagnosis. Length of antennae less than half that of costa, with apiculus small and bent. Third segment of palpi short and bent slightly forward. New genus differing from other genera of tribe Baorini by harboring bifid uncus and developed socius.

Etymology. The genus is named after the late Dr. Torben Larsen, the leading expert on African butterfly taxonomy, who was a member of this project. He passed away suddenly in May 2015 and therefore did not see the final results of this research; with respect, we would like to name the new genus after him.

In our analyses, four species currently treated as members of the genus *Borbo*, namely *B*. *gemella*, *B*. *perobscura*, *B*. *holtzi*, and an unidentified species formed a distinct group that is basal and sister to the rest of Baorini. Based on these results, we established *Larsenia* as a new genus. Before describing *Borbo*, Evans [19] divided brown skippers into *Baoris* and *Pelopidas*. The three species above were all assigned to *Pelopidas*. After describing *Borbo*, he divided members into two groups: one with smooth mid-tibia and the other with spined mid-tibia [79]. Both *B*. *perobscura* and *B*. *holtzii* have spined mid-tibia but not *B*. *gemella*. These three species are autapomorphous with respect to their male genitalia, with developed socius. Although it is beyond the scope of this study, a detailed description of the new genus is in preparation pending further research determining which members of the African *Borbo* that were not included in this study should be assigned to the new genus.

### *Pseudoborbo* Lee, 1966 confirmed status

**Fig 2:**
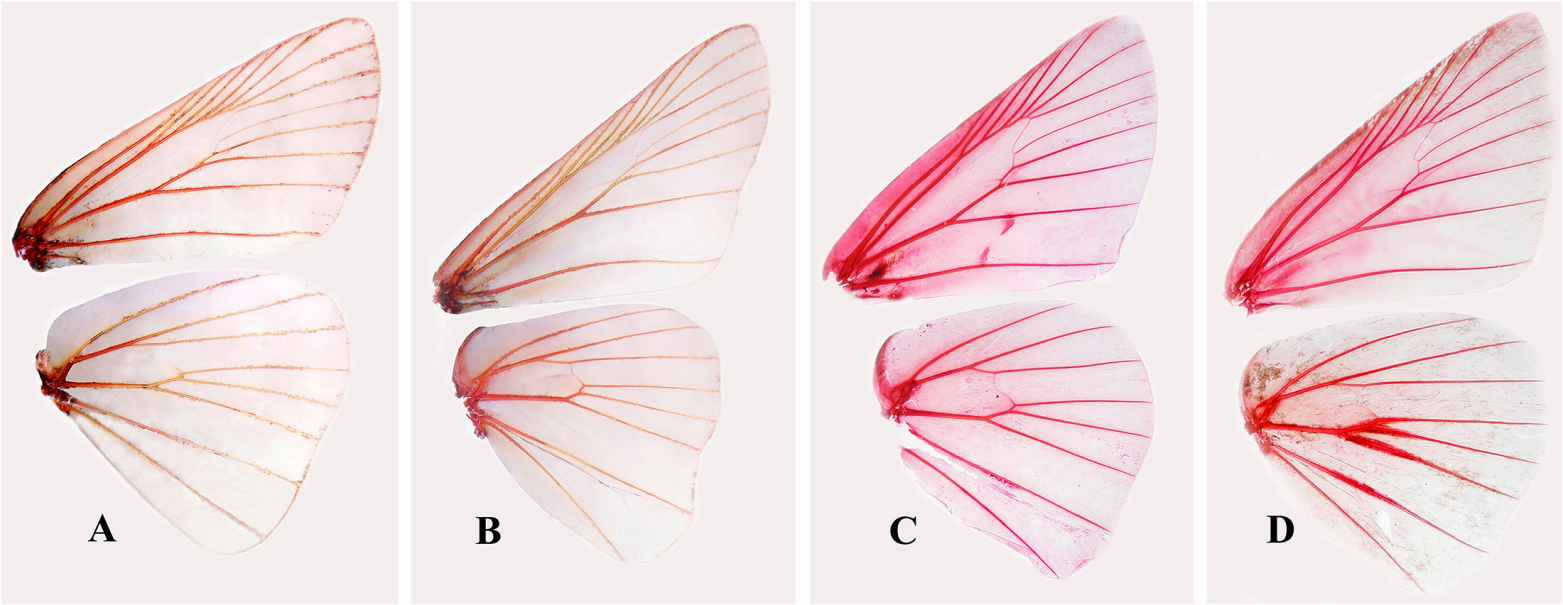
Wing venation of Baorini. (A) *Pseudoborbo bevani* (Moore, 1878); (B) *Borbo borbonica* (Boisduval, 1833); (C) *Zinaida nascens* Leech, 1893; (D) *Polytremis lubricans* (Herrich-Schäffer, 1869).

**Fig 3:**
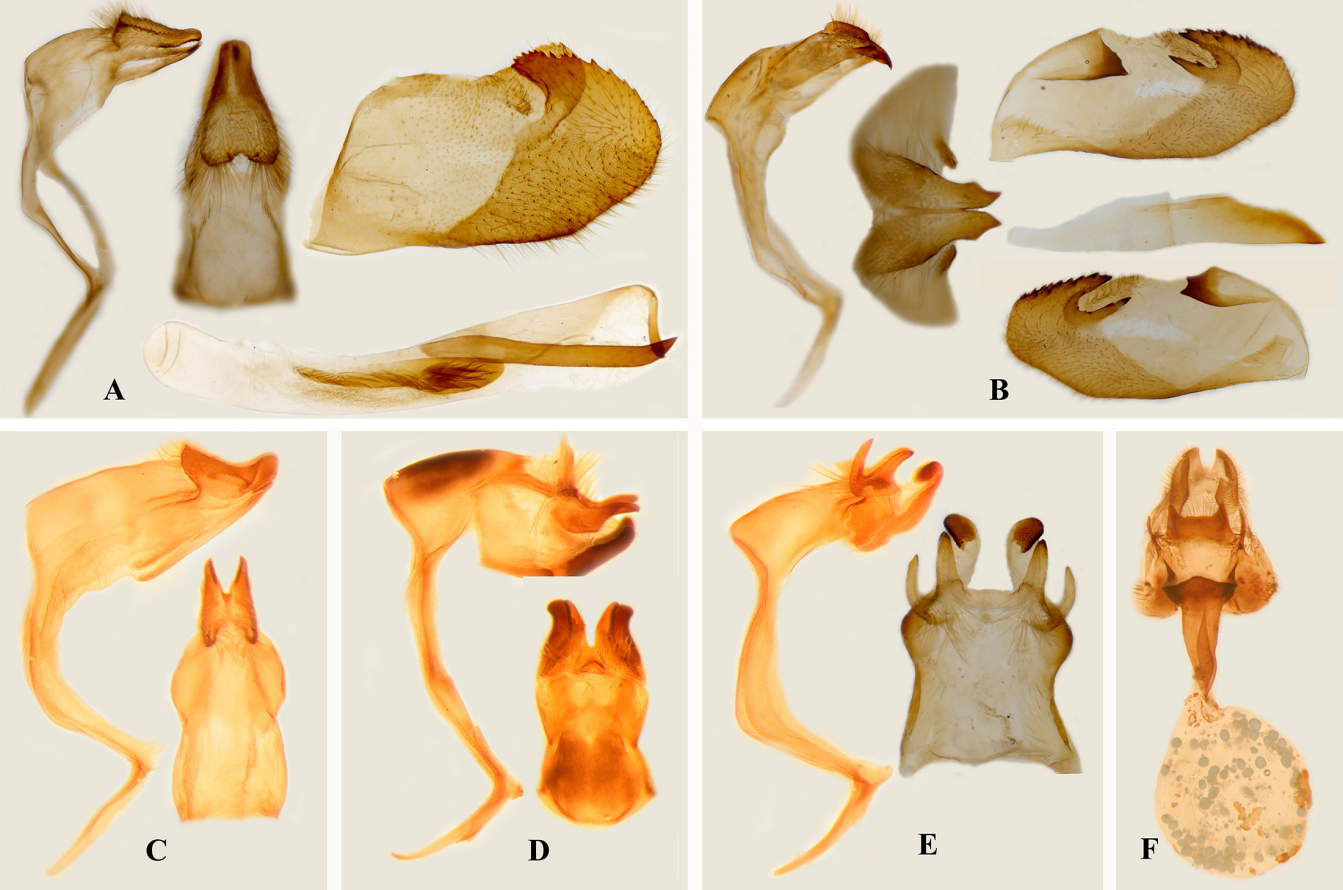
Genitalia of Baorini (A-E, male; F, female). (A) *Pseudoborbo bevani* (Moore, 1878); (B) *Borbo borbonica* (Boisduval, 1833); (C) *Zinaida nascens* Leech, 1893; (D) *Zenonoida elota* (Hewitson, 1869); (E & F) *Polytremis lubricans* (Herrich-Schäffer, 1869).

Our morphological study shows that the type species of both genera are greatly different in wing venation and male genitalia. Specifically regarding wing venation (Fig 2A and 2B) on the forewing, the origin of M_3_ is branched midway between M_2_ and CuA_1_ while on the hindwing, the origin of vein CuA_1_ is distinctly closer to M_3_ than to CuA_2_ in *Pseudoborbo*. Simultaneously, on the forewing, the origin of the vein M_3_ is distinctly closer to M_2_ than to CuA_1_, and on the hindwing, the origin of vein CuA_1_ is branched midway between M_2_ and CuA_2_ in *Borbo*. In the male genitalia (Fig 3A and 3B) of *Pseudoborbo*, the uncus not separated at tip, while the gnathos is developed and nearly reaches the tip of uncus; the valva lacks transtilla, and the aedeagus is characterized by a thick, long spine and an uneven cornuti. However, in *Borbo*, the uncus is bifid and bent ventrally at the tip, the gnathos is far from reaching to tip of uncus, the valva harbors transtilla, and the aedeagus is simple without cornuti. Eight species of traditional *Borbo*, including the type species *Hesperia borbonica* Boisduval, 1833, as well as the type and sole species of *Pseudoborbo*, were analyzed in our molecular study. The results revealed that *Pseudoborbo bevani* is located separately from the two clades of the other members of *Borbo*. The relationship of *P*. *bevani* to the sister clades *Pelopidas* and *Baoris* is closer than its relationship to *Borbo*. Morphologically, *Pseudoborbo* is also much more similar to *Pelopidas* and *Baoris*, especially with regard to the male genitalia.

Based on molecular evidence as well as morphological characters, we propose that the genus *Pseudoborbo* Lee, 1966 is valid.

### *Borbo* Evans, 1949

Currently, the genus *Borbo* consists of five Indo-Australian and 18 African species [22]. These species vary extensively in the morphology of the male genitalia, and, therefore, it is necessary to divide them into several groups according to their characteristic genitalia structures [78]. Our analyses clearly indicate that the eight species analyzed here are polyphyletic. Although *Borbo*, excluding Clade A and *Pseudoborbo*, forms a moderately supported clade, the relationship among the three sublineages (*B*. *cinnara*, *B*. *borbonica*, and *B*. *fatuellus+B*. *ratek*) is unclear. We did find that *B*. *fatuellus* is sister to *B*. *ratek* and each sublineage differs according to male genitalia morphology. Evans [19] determined that *Baoris* included *B*. *ratek* and *B*. *fatuellus* and *Pelopidas* included *P*. *borbonica*. Again, mid-tibial characteristics do not appear to be informative, since *B*. *ratek* and *B*. *fatuellus* have smooth mid-tibia while *P*. *borbonica* has a spined mid-tibia. However, since the sample size is not sufficient and the support for the *Borbo* clade is relatively low (PP = 0.83, B = 70), additional species sampling and gene sequencing are necessary to resolve the phylogeny of *Borbo* in the future.

### *Prusiana* Evans, 1937 confirmed status

*Prusiana*, a small genus with only three species, is obviously a monophyletic group with a synapomorphy in which the males have a brand at the base of the space M_1_ on the hindwing [6, 22]. Nevertheless, the taxonomic position of *Prusiana* has been controversial, as mentioned above. Based on morphology rather than molecular evidence, Warren *et al*. included *Prusiana* in Baorini [2]. The molecular phylogeny presented here clearly indicates that *Prusiana* is a member of Baorini and that its sister-group relation to *Catoris* is weakly supported in the BI phylogeny (PP = 0.79, BS = 38).

### *Polytremis* Mabille, 1904

In our present analyses (Fig 1, S1 Fig), twelve species of *Polytremis*, sensu Evans [6], were not determined to be a monophyletic group but were split into three strongly supported and very distant clades, of which the clade with the type species *P*. *lubricans* harbors five representative individuals from China and Malaysia. Therefore, we now recognize *Polytremis* Mabille, 1904 to be a monotypic genus (type species *Goniloba lubricans* Herrich-Schäffer). Morphologically, the genus is distinguishable based on the male genitalia (where the lateral process of the uncus, which is divided and horn-like, is clearly separated at its base (Fig 3E)) and the female genitalia (with sclerotized fingerlike projections between the anterior and posterior lamella (Fig 3F)).

### *Zinaida* Evans, 1937 reinstated status

Our morphological study shows that *Zinaida* is quite different from *Polytremis* in wing venation and genitalia. Unique characteristics in wing venation in *Zinaida* (Fig 2C) include the forewing, in which the origin of R_1_ follows that of CuA_2_ and is located nearly midway between CuA_1_ and CuA_2_, and the hindwing, in which the origin of Rs is before that of CuA_2_. However, in *Polytremis* (Fig 2D), the origin of vein R_1_ is opposite CuA_2_ and the origin of Rs is opposite CuA_2_. In addition, males of most species have a stigma in space CuA_2_ on the upper side of the forewing, and in *Polytremis* males, the hindwing expanded at middle A, basal M_3_, CuA_1_, and CuA_2_. The male genitalia (Fig 3C and 3E) in *Zinaida* are unique since the uncus is V-shaped, projects at the left and right and is attached at its base, while the gnathos is straight and has an attached uncus. In *Polytremis*, the uncus is completely separated, and the gnathos is elbow-shaped and located far from the uncus.

Of the 18 species included in *Polytremis* sensu Evans [6], 12 species, including the type species of both *Polytremis* and *Zinaida*, were analyzed in our study. Three clades were defined using all methods. One clade consisted of five individuals of *P*. *lubricans*; *P*. *discreta* and *P*. *eltola* and formed a strongly supported clade (PP = 1.00, BS = 100, BP = 100), which is sister to *Zenonia* with moderate support (PP = 0.93, BS = 70, BP = 66). The other samples, including *P*. *nascens*, formed a strongly supported monophyletic group. Our study thus suggests that the monophyly of *Polytremis* presented by Evans should be rejected and the genus *Zinaida* reinstated. Our result contradicts that of Jiang *et al*. [55]. In their analysis, the monophyly of the genus *Polytremis* is weekly supported in ML analysis (BS = 52 on the concatenated data; and BS = 73 on *COI* sequence), even though they claim that the monophyly is strongly supported. On the other hand, the clade including *P*. *lubricans*, *P*. *eltola*, and *P*. *discreta* is strongly supported (BS = 99 for both the *COI* sequence and combined data set). The DNA markers and samples (ingroup and outgroup) selected are essentially why the results are different. First, they used one mitochondrial gene *COI* (490 bp) and two nuclear genes (the D3 region of *28S* rRNA gene and the V4 and V7 regions of the *18S* rRNA gene, in total 1048 bp). The trees derived from the separate analyses of *COI* as well as the concatenated sequences (*COI*+rDNA) have roughly similar topologies; however, we determined that the *COI* gene contributed more to the phylogenetic signal, and combined analyses yielded lower resolution. This is because the two slowly evolving rDNA genes are usually used in higher taxonomic levels studies [80, 81]. Additionally, different genes are phylogenetically informative at various taxonomic levels [82]. Therefore, choosing suitable genetic markers is a key element in reconstructing improved molecular phylogenies. We chose *COI-COII* and *16S rRNA* from mitochondrial DNA, rDNA *EF-1α*, and *28S rRNA* as molecular markers. All of these markers have been previously used successfully to elucidate the relationships among many groups within the Lepidoptera, including at the levels of genera, tribe, and subfamily [5, 57, 82–89]. Second, 15 Chinese species were used as the ingroup and four Baorine genera as the outgroup. Despite the relatively large number of samples included in the ingroup, the result of molecular phylogeny analysis is not ideal due to the unsuitable outgroup. Since relationships among genera in Baorini are unclear and *Polytremis* is a morphologically diverse group, all available genera should be included as the outgroup in analyses instead of only four. Our study included nearly all the major genera within Baorini all over the world. In order to test previous analyses, our study included 12 species, allowing for a broad representation of lineages within *Polytremis*, and containing more than three individuals for *P*. *lubricans*, *P*. *eltola*, and *P*. *discreta*. Although our species sampling is less extensive than in previous studies, the present trees (Fig 1, S1 Fig) are better resolved than those from Jiang *et al*. [55] and reveal that that *Polytremis* sensu Evans [6] is not a monophyletic group, *P*. *eltola* as sister group to *P*. *discreta* rather than to *P*. *lubricans*.

### *Zenonoida* Fan and Chiba gen. nov.

urn:lsid:zoobank.org:act:8CA5AEF0-E81D-4F74-8CA1-F62C407A5FBA

#### Type species. *Hesperia eltola* Hewitson, 1869 (Male)

Diagnosis. New genus superficially similar to *Polytremis* Mabille, 1904 and *Zinaida* Evans, 1937, though distinguishable from other two genera as follows: palpi characterized by short third segment, stout and barely protruding; forewing cell spots conjoined or upper cell spot absent. Uncus with central-basal area membranous; gnathos elbow-shaped, sclerotized except for a narrow distal membranous band.

Etymology. The scientific name, *Zenonoida* is derived from the genus *Zenonia* since the new genus is significantly similar to *Zenonia* with respect to the male genitalia.

In our analyses, *P*. *eltola* and *P*. *discreta* were assigned to *Polytremis* sensu Evans [6], which is distantly located from both *Polytremis* and *Zinaida*. Thus, we describe *Zenonoida* as a new genus, and move *P*. *elota* and *P*. *discreta* from *Polytremis* sensu Evans [6] to the new genus: *Z*. *elota* com. nov., *Z*. *discreta* comb. nov.

## Supporting Information

S1 Fig Strict consensus cladogram of the 5 equally parsimonious trees (length 3456, consistency index 0.402, retention index 0.679) inferred in TNT analysis of the concatenated *COI-COII*, *16S*, *EF-1α* and *28S* sequences. The numbers indicate bootstrap values. Colors highlight recognized genera.
(TIF)
