# Supplementary figures and images for: Clarification of the Phylogenetic Framework of the Tribe Baorini (Lepidoptera: Hesperiidae: Hesperiinae) Inferred from Multiple Gene Sequences
Source: PLoS One. 2016 Jul 27;11(7):e0156861. doi: 10.1371/journal.pone.0156861 (PMC4963138; doi:10.1371/journal.pone.0156861)

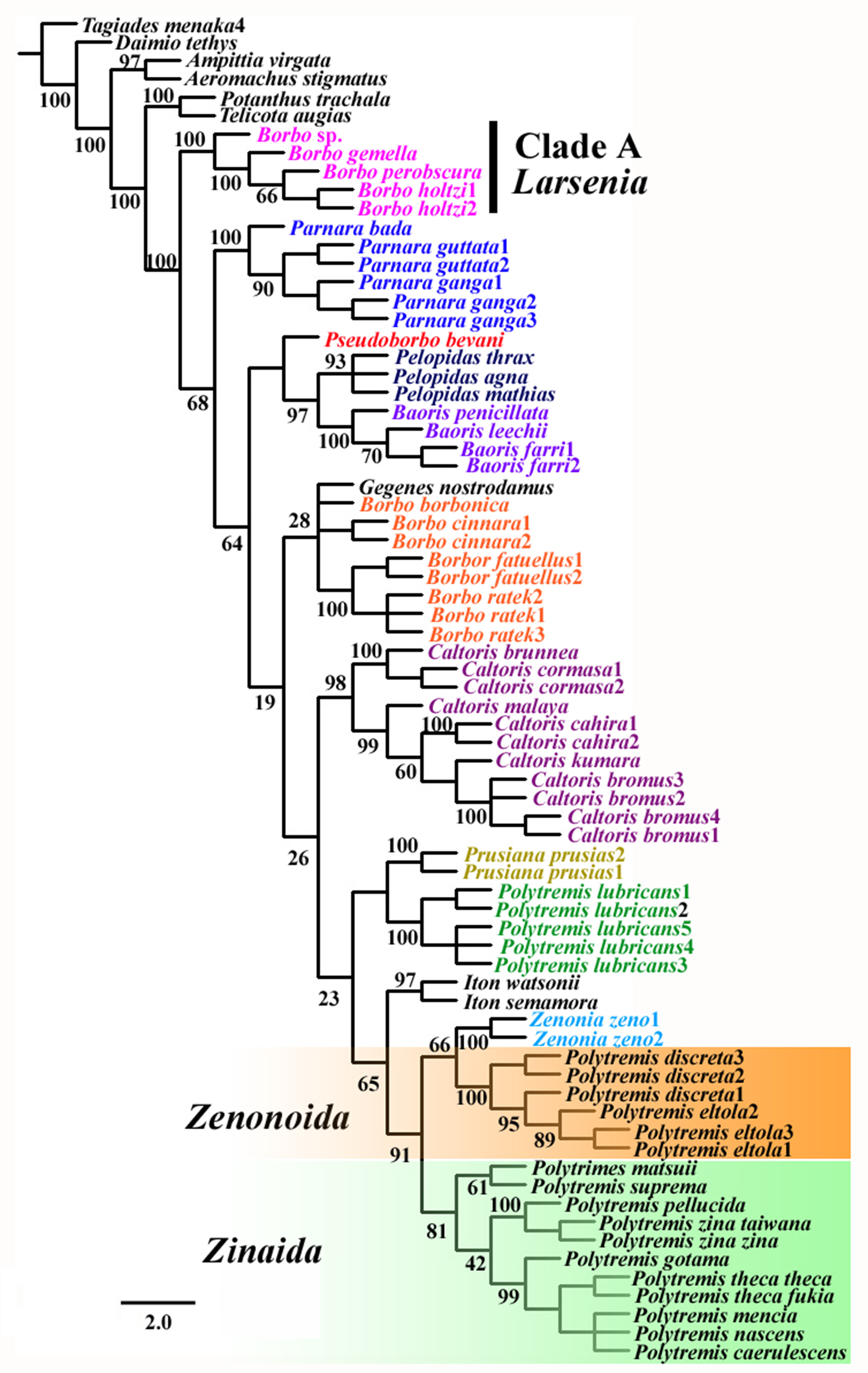

Supplement: S1 Fig — The numbers indicate bootstrap values. Colors highlight recognized genera. (TIF) [file pone.0156861.s001.tif]
